# MAGPIE: Simplifying access and execution of computational models in the life sciences

**DOI:** 10.1371/journal.pcbi.1005898

**Published:** 2017-12-15

**Authors:** Christoph Baldow, Sebastian Salentin, Michael Schroeder, Ingo Roeder, Ingmar Glauche

**Affiliations:** 1 Institute for Medical Informatics and Biometry, Medizinische Fakultät Carl Gustav Carus, Technische Universität Dresden, Dresden, Germany; 2 Biotechnology Center (BIOTEC), Technische Universität Dresden, Dresden, Germany; 3 National Center for Tumor Diseases (NCT), Partner Site Dresden, Dresden, Germany; Universite de Montreal, CANADA

## Abstract

Over the past decades, quantitative methods linking theory and observation became increasingly important in many areas of life science. Subsequently, a large number of mathematical and computational models has been developed. The BioModels database alone lists more than 140,000 Systems Biology Markup Language (SBML) models. However, while the exchange within specific model classes has been supported by standardisation and database efforts, the generic application and especially the re-use of models is still limited by practical issues such as easy and straight forward model execution. MAGPIE, a Modeling and Analysis Generic Platform with Integrated Evaluation, closes this gap by providing a software platform for both, publishing and executing computational models without restrictions on the programming language, thereby combining a maximum on flexibility for programmers with easy handling for non-technical users. MAGPIE goes beyond classical SBML platforms by including *all* models, independent of the underlying programming language, ranging from simple script models to complex data integration and computations. We demonstrate the versatility of MAGPIE using four prototypic example cases. We also outline the potential of MAGPIE to improve transparency and reproducibility of computational models in life sciences. A demo server is available at magpie.imb.medizin.tu-dresden.de.

This is a PLOS Computational Biology Software paper.

## Introduction

**Fig 1:**
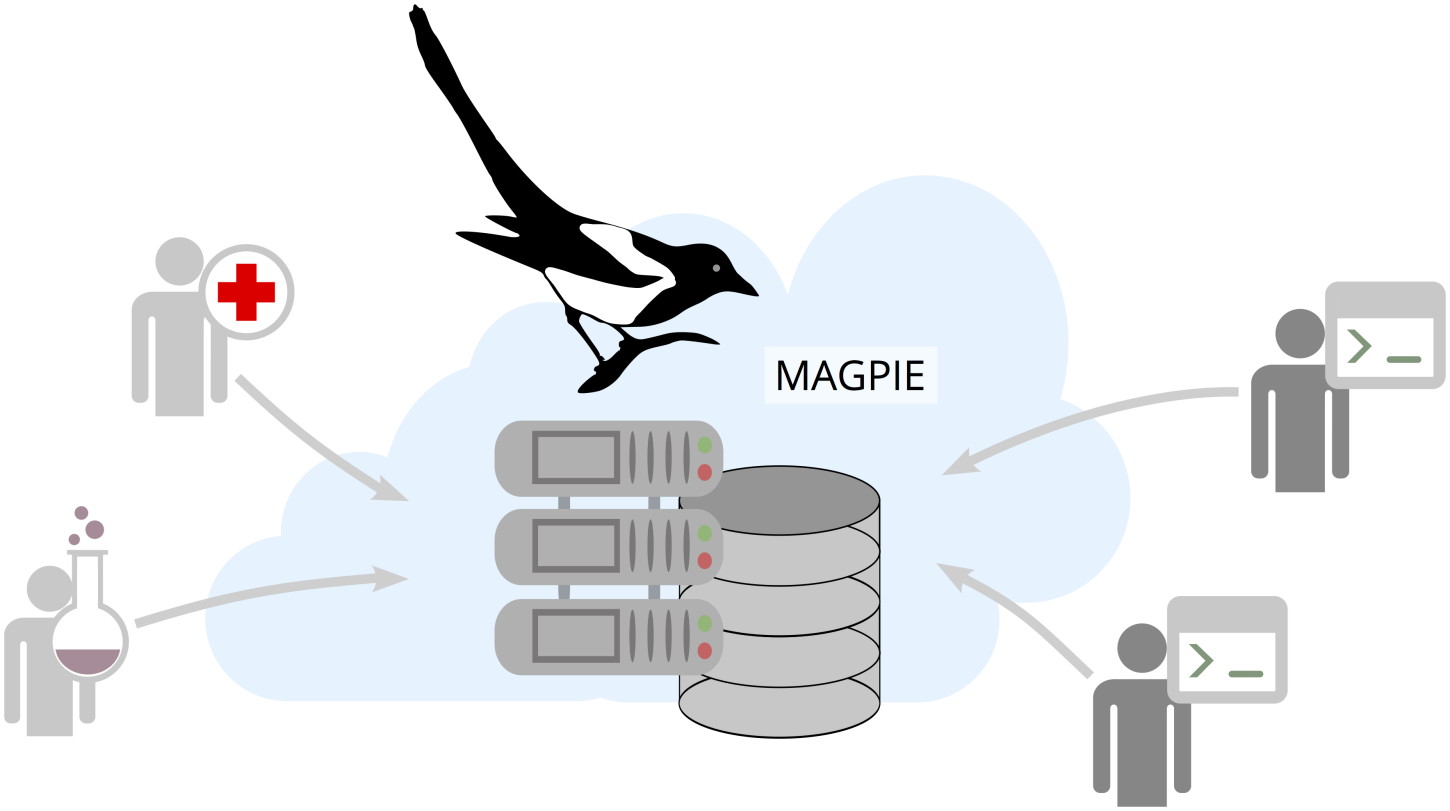
Collaborative research with MAGPIE. Physicians and biologists (left) can use models provided by computational experts (right) to analyse their data in MAGPIE (center). The central element of the platform comprises a computing framework and a model database.

Although the development and use of computational models is gaining more and more importance in the era of biological and big data, there is a distinct gap between the development of computational models by theoreticians and their application by end users in experimental research or clinical practice [1–3]. In many cases, technical issues limit the application of computational approaches: models commonly lack an easily accessible user interface or require advanced programming skills on the side of the end user. Furthermore, the limited access to models, or missing versioning of underlying algorithms, data, and parameters lead to model developments, which in many cases can not be easily reproduced [4, 5]. In a recent survey conducted by Nature, more than half of the participating researchers believed that there is a significant crisis in model and data reproducibility [6]. Our platform MAGPIE addresses this issue by providing a single platform where researchers from different domains can publish, exchange and execute models on-line (Fig 1) as well as publish and exchange data.

In the past, there have been several approaches to provide a unified platform for computational models in the biological domain: Standardised description languages for models, foremost SBML (Systems Biology Markup Language) [7, 8], but also CellML [9], NeuroML [10] or MultiCellDS [11] and associated model databases such as BioModels [12] provide platforms to store and exchange standardised models. The Cardiac Electrophysiology Web Lab [13] is a further example of a database for standardised models, which allows to compare model behaviour under different conditions. Such tailor-made approaches for the description of specific processes are valuable for specific domains, but impose restrictions on other applications and their interoperatibility. Going one step further, workflow tools such as Taverna or GALAXY [14, 15] are application-oriented and help the user to integrate multiple analysis steps. Here, it is possible to combine SBML models, custom scripts, and web services. While this approach is powerful, the platforms themselves bring a high overhead when it comes to simple applications and may be unintuitive for inexperienced users. On the other side of the software spectrum, social networks for scientists, e.g. ResearchGate or Mendeley, aim to make individual research more visible by allowing the exchange of publications and expertise. However, they do not currently offer to share and publish data-driven analyses or modelling approaches.

The main objective of MAGPIE is to provide a user-friendly system to publish, version, access and execute models in an environment that is accessible for different end users. Contrary to the idea of developing a new modelling standard, different types of computational models can be uploaded with minimal preparation. While MAGPIE also supports SBML, it puts no restrictions on programming languages or frameworks. The focus for model execution is not on complex workflows, but on *one-click execution* of single models. This makes it easy for end users to apply algorithms that have been previously deposited by computational experts. A hashtag-based system allows to share results and projects with other users.

The central layers of MAGPIE are a repository of models and analysis tools, a project-oriented access to the models that allows changing parameter configurations and data input, program execution, visualization of results, as well as a twitter-like hashtag system to simplify the sharing of results increasing the visibility of individual researchers. Automated version control, remote access, and virtualization are available by default.

## Design and implementation

### MAGPIE server

MAGPIE was implemented in *Ruby on Rails 5*. The public demo server is based on an *nginx* [16] environment in combination with *foreman* [17], *Phusion Passenger* [18], and *Sidekiq* [19] for simplified application management. Furthermore, it allows flexible scaling of MAGPIE for local installations and distributed cluster environments. The demo server uses *PostgreSQL* [20] as a database backend system.

### Security and virtualization

MAGPIE ensures security features such as separateness, reproducibility and computation security, thereby allowing to be applied in systems medicine and potentially in clinical practice. It is implemented in a privacy-oriented fashion, such that everything a user wants to share has to be published explicitly. Additionally, MAGPIE relies on Docker [21] virtualization for executing jobs using *docker-api* for Rails. This brings the advantage of separateness and at the same time it can be ensured that a model can not harm the host system (e.g. the web server itself). The Docker containers also provide lightweight virtual model execution environments. Administrators of MAGPIE can install components and programming languages in these containers to be used by the models or completely exchange the execution environment. Additionally, they can use a role-based access control system.

### Version control

The storage of models and corresponding data is organized by the repository system *git* to enable full reproducibility of calculated results. Whenever a new model is uploaded or an existing model is updated, a new *git* repository is created or the existing model is updated with a new commit. During project creation, the model as well as the version have to be defined by the user. Furthermore, the execution environment itself is versioned. Whenever a new project is created, the actual version of the execution environment is linked to the project, such that the identical execution environment is available for further job executions.

### Environment and programmatic access

The MAGPIE source code can be downloaded from the GitHub repository at https://github.com/christbald/magpie and installed on any public or private server, as well as on any local computer.

Additionally, we offer an R package at (github.com/christbald/magpie_api_r) to provide a *power user gateway* to MAGPIE. The R package has been developed to provide remote access for creating projects and jobs, and subsequently accessing the results. Furthermore, the package can be integrated with other applications, such as RStudio’s Shiny: this makes MAGPIE a potential backend for asynchronous job executions enabling non-blocking applications with cloud computing.

## Results

### MAGPIE models

**Fig 2:**
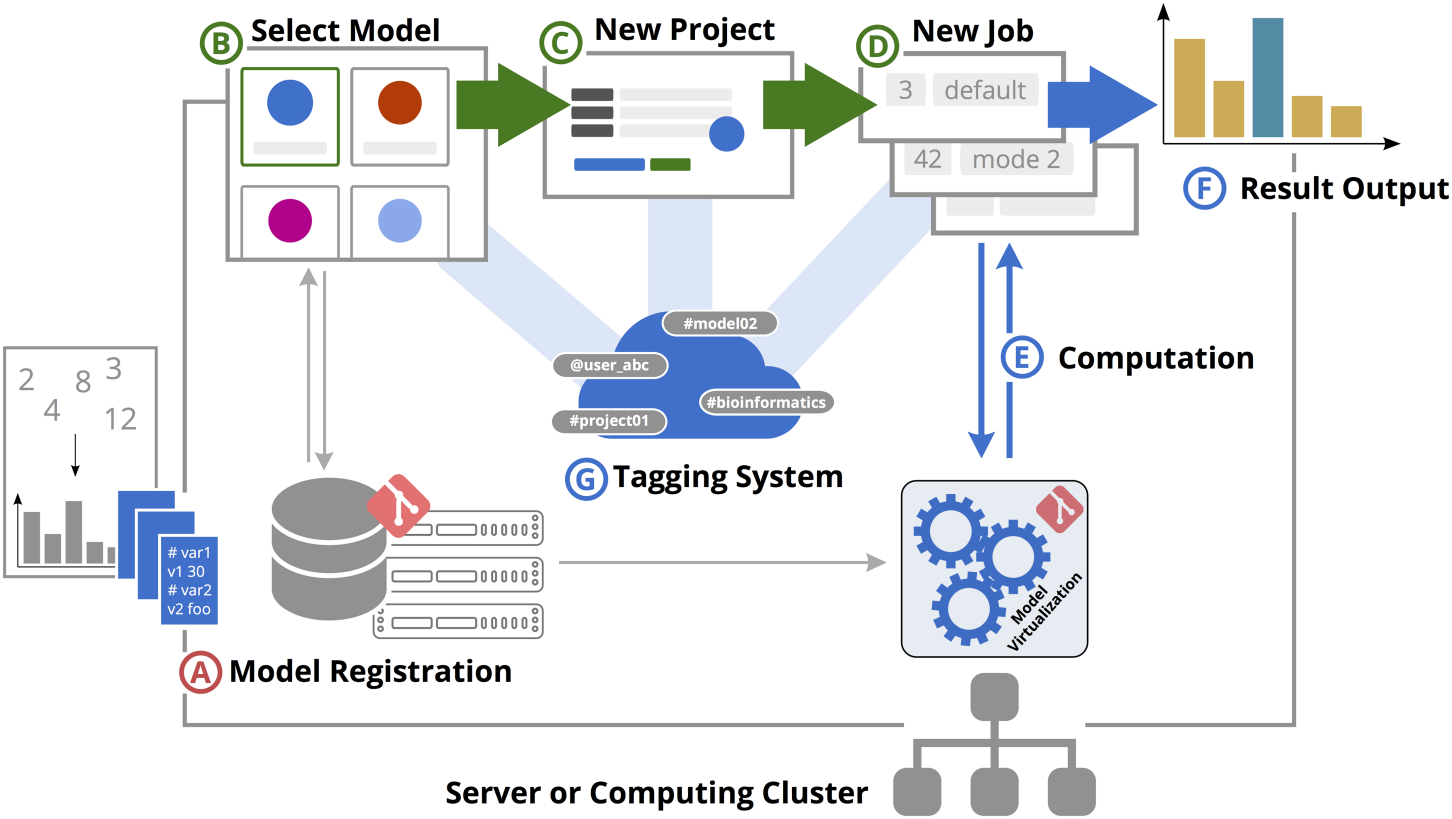
The MAGPIE workflow. After models are registered on the platform (A), they are available to any user (B) for the use in analysis projects (C). A project organizes single jobs (D), which enable to run computations with the selected model and different parameter sets on the server (E). Results can be directly viewed in the browser (F). Projects and models of interests can be tagged and shared with other users (G). Steps marked with letters in red color are performed by modellers (A). Other users (green) have simplified access to the organization of projects, jobs, and results (B,C, and D). Computation in Docker containers, interactive result output, and organization by tags (blue: E, F, and G) is handled in the background by MAGPIE.

MAGPIE has been designed as a software platform for both, publishing and executing computational models. New models and analysis tools can be easily registered to MAGPIE within the browser (see Fig 2A) to make them available to other users. The web server allows for models in any programming language along with a shell script as entry point and an adaptable parameter file. The shell script starts the simulation in a unix environment while the parameter template defines the names and types (numeric, conditional, file upload, etc.) of variables in the model. MAGPIE uses this template to generate an input form, which is visualized in the browser for every new job. Upon job execution, a parameter file instance is provided to the model reflecting the particular user configuration.

### Workflow

From the user perspective, a *project* is defined by a predefined *model*, including the version and an execution environment. Furthermore, it contains *jobs* (single model instances), which are defined by a set of parameters defined in the corresponding model. All information about projects, jobs, and their results are stored in a database.

A typical application-oriented procedure, i.e. the use of MAGPIE by a fellow biologist or medical researcher presents as follows: The user chooses from the available models (Fig 2B) and starts a new project, which is now linked to the model. A project will typically contain a series of computations (*jobs*) on the same model with different parameter sets. For each computation, a job is initiated on the server (Fig 2D) and automatically processed in the background (Fig 2E). The user is notified when a job is finished and can immediately view the results in the browser (Fig 2F). Projects and models of interest can be tagged, shared with other researchers and discussed via the integrated messaging service (Fig 2G).

### Applications

**Fig 3:**
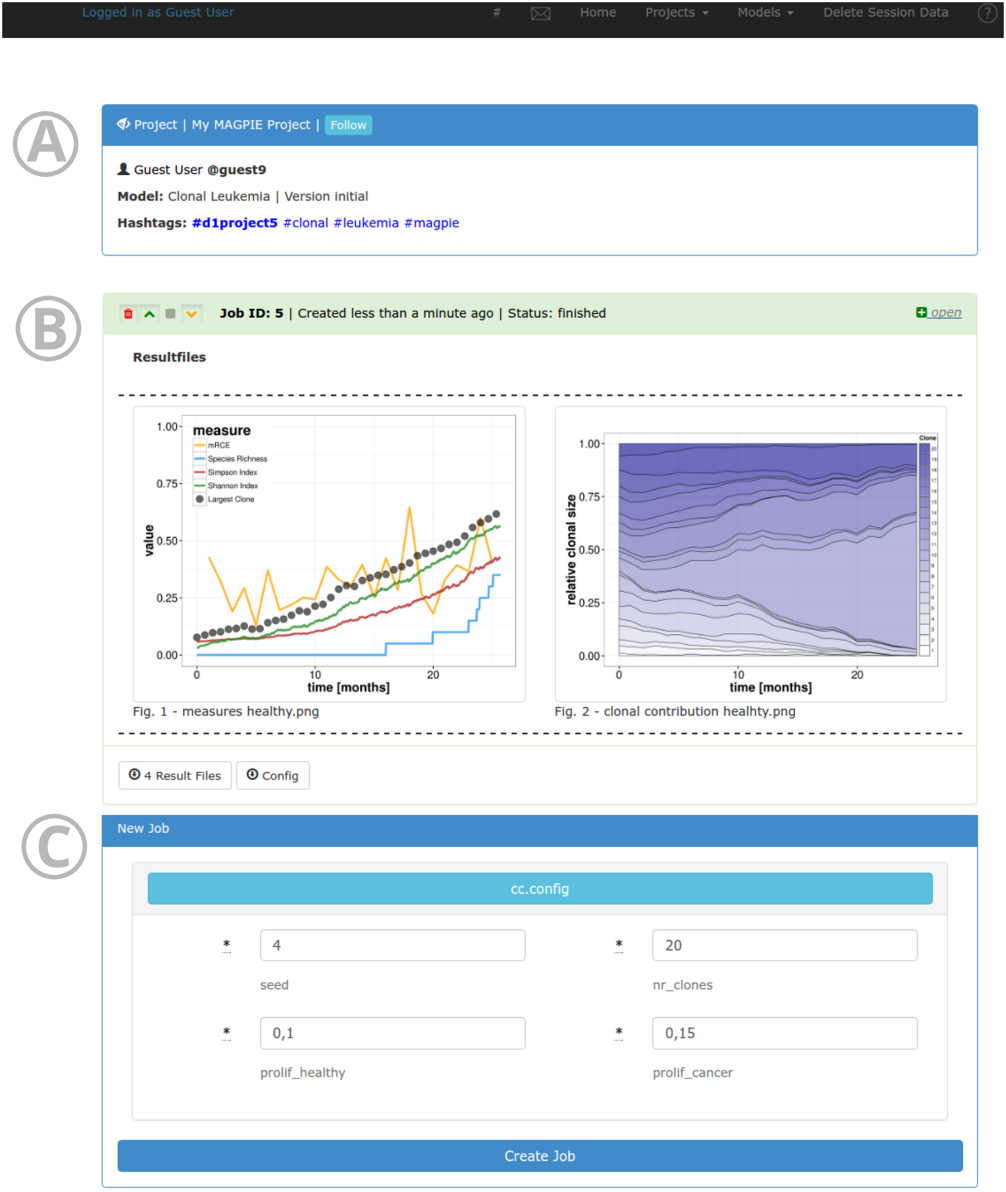
Project page in MAGPIE. The header (A) shows general information on the project, such as the owner, title, version, and associated tags. The status of jobs and their results can be directly viewed in the browser (B). All output files are rendered on the page and are available for download. A new job with different parameters can be also started from the project view with one click (C).

The MAGPIE demo server is equipped with several test models. *Clonal Leukemia* is a single cell based model implemented in R focussing on the impact of different population-based properties to influence competition between healthy and cancer cells [22]. After creating a project (Fig 3A), a new job with corresponding parameters can be requested (Fig 3C). MAGPIE executes the model and displays the graphical output on the temporal outgrowth of cancer cells (Fig 3B). This model serves as a prime example for the ease of model integration in MAGPIE and shows the flexible support for widely used programming languages such as R.

A more comprehensive integrated example model is the structural bioinformatics toolbox *PLIP* [23]. PLIP is written in Python and serves as a prototypic example from the structural bioinformatics domain. It allows to analyse molecular contacts between a protein and its ligands from a crystal structure file (e.g. PDB files). All major parameters from the published command line tool have been integrated. Advanced form fields allow to switch between the input of a structure ID (PDB ID) or the upload of a structure file to the server. If a valid PDB ID is given, the structure file is automatically fetched via a REST service from the external PDB server. The generated interaction diagrams can be viewed directly in the browser or downloaded for local processing. An interactive plot was added to compare contact distances between different binding sites of a protein. This example shows that an existing tool without a GUI can be made accessible to a larger user group using MAGPIE.

A third example, named *Multiplexing Clonality* [24], is a R-based tool to analyse raw sequencing data from barcode experiments. This model focusses on a statistical evaluation given a particular set of data. Here, the end users are able to change parameters of the given statistical analysis, which is then applied to the sequencing data from the corresponding publication. The data is uploaded to MAGPIE along with the analysis algorithm. This model illustrates the integration of internal data sources, showing that MAGPIE goes far beyond a modelling executing platform.

Furthermore, we integrated a fourth model, studying the enhancement of *STAT1* in pancreatic cancer [25], published already on BioModels as an SBML model. This showcase demonstrates the ease of SBML integration, and it documents the workflow when integrating, and optionally modifying SBML models. In brief, users can transfer an SBML model directly from the BioModels database. In this case, a corresponding MAGPIE compatible parameter file is automatically created from the SBML input using Python. Additionally, the C-based SBML execution environment LibSBMLSim [26] and the visualizing script based on R are automatically integrated into the newly created MAGPIE model.

Optionally the generated SBML model and the parameter file can be exported, modified and re-uploaded to obtain a customized version of the model. Similar to SBML, MAGPIE can be further extended to support other standard formats.

Additional showcases do highlight further features of MAGPIE. Based on these, users can acquaint themselves with different aspects of MAGPIE, e.g. interactive graphical plots, entries in the parameter templates, and error handling.

### Simplicity

MAGPIE stands out by providing a clean interface for organizing reproducible research. The main menu provides access to the model repository, the project pages with results of computations, and the user profile. New computations can be started with one click, but if necessary also provide access to the full parameter set of the associated model. For users on the platform, no technical knowledge on the models is necessary: they can start computations with a simple input form for parameters directly in the browser. The request is handled automatically in the background and results are visible directly after the job is finished (Fig 3B). For modellers, MAGPIE also provides a simple system to register new algorithms, which are then immediately available to the scientific community.

All websites are equipped with a context sensitive help menu, i.e. a help button leads directly to the corresponding help menu entry. Thereby, we ensure our simplicity paradigm to the highest level.

### Flexibility

MAGPIE is not restricted to certain script languages or a predefined set of programming languages in contrast to other platforms, like GALAXY and Taverna. Each unix-executable program can be registered. The flexibility is ensured by the prerequisite to provide a small wrapper (shell) script for program execution and a generic configuration file to define model parameters presented to users on the platform. A detailed documentation on the preparation of models is available on the public demo web server. Additionally, MAGPIE supports models in the widely-used Systems Biology Markup Language (SBML) [27], which enables the execution of thousands of available models from the BioModels database [12] directly in MAGPIE. For modellers, MAGPIE offers rapid publication of scripts and algorithms, which can be directly accessed and run on a public or private server by other users without any technical setup.

Project-oriented access to the models allows to change parameters and/or input data within the automatically generated graphical user interface and to immediately execute the simulations/analyses within the MAGPIE environment.

In addition to the publicly available demo version of MAGPIE, the complete MAGPIE web application can be downloaded from the demo server, customized and installed in any environment. Limitations on available computational resources as well as regulations on model upload policies can be implemented by the local MAGPIE administrator as needed. For power users, MAGPIE can be accessed programmatically via an API and thus be used as a backend for other (server) applications. An R package for the API is provided at github.com/christbald/magpie_api_r and allows to create and organize projects or jobs and to retrieve results from individual computations.

### Reproducibility

MAGPIE automatically records used model versions and model environments in the background. Furthermore, it tracks all changes from model updates and enables users to go back in time and select all previous versions of models to run computations. All parameter sets from jobs are stored alongside the results and are available for download. This is an essential aspect of reproducible research in computational biology, and it makes MAGPIE an ideal model repository for research journals that aim at facilitating broad access to data, reproducibility of models, and easy re-evaluation of published analyses. Furthermore, the high standards for reproducible model execution position MAGPIE as a potential interface towards clinical applications.

### Connectivity

MAGPIE provides the option to share and announce new models, projects, results and feedback via a Twitter-like messaging system. In MAGPIE, *hashtags* can be assigned to models, projects, or messages. Users can define which topics they are interested in and retrieve updates about projects or researchers of interest. Updates and messages are integrated in a single messaging system available in a sidebar on the website.

The model output of a job is directly displayed and amenable to further processing by automatically creating a download link to all model configuration and output files. This structure allows using MAGPIE as a platform for collaborative efforts in systems biological approaches involving peers from various domains and with different computational skills. On a bigger scale, potentially even multiple MAGPIE server instances could be linked to form a decentralized model execution repository of various groups and communities.

### Standardization

In the modelling community many different tools and individual preferences for particular implementation exist and arise. To ensure usability across groups many efforts were applied to counteract a further fragmentation. The development of standardized formats is one potential strategy which requires continuous adaptation to the needs of the modelling community. Therefore, different standards were developed with different rationales [28]. As a result, model representation and model simulations are often decoupled, leading to a better formalisation and comparability of different models. As a prominent example, SBML is a markup language for the description of biological systems. Although the standardization allows for intuitive visualizations and easier coupling of models, the model execution requires a particular model environment and is not trivial to access for many non-technical users.

In contrast, MAGPIE does not focus on standardization. Although it supports standardized formats (e.g. SBML), MAGPIE provides much more flexibility to the model developers, thus lowering the barriers to deposit and run models online. Additionally, for publishing with MAGPIE the translation into another scripting or markup language is not necessary, thereby reducing the sources of error. Like JWS, an online SBML execution environment [29], MAGPIE provides the opportunity to modify, update and share existing SBML models. At the same time MAGPIE can cover complex models as well as models from novel research domains for which no standards are yet established.

## Availability and future directions

### Perspective

The importance of computational models in application oriented science, such as biomedicine, has rapidly increased over the last years. However, there is still a level of reluctance towards model application by end users such as “wet lab” biologists or clinicians. These problems were already identified in the seminal works of Star and Griesemer in the late 80s [30], who suggested the concept of *boundary objects* as a shared space of information for different communities. Next to its organizational structure (*material*), a boundary object is defined by an interpretive flexibility and a certain level of abstraction (*scale* or *granularity*), which enables accessibility by different communities [3]. On this conceptual level, MAGPIE serves as a boundary infrastructure comprising a collection of boundary boxes, which are developed to connect users from different domains. Although we focused on biological sciences the concept of MAGPIE can be extended to other fields of research in a straight forward manner. In order to preserve the funtionality as a boundary object, MAGPIE’s further developments need to involve all target communities in an iterative process.

In a proof-of-concept study with clinical collaborators, MAGPIE is already used as a demonstrator for pre-clinical applications in the context of predicting minimal residual disease levels in leukemia. We are envisaged to start more user-oriented projects in different scientific communities to acquire new models and further improve the usability of MAGPIE.

Direct applications of MAGPIE extend even beyond the domain-bridging efforts. Especially research journals and publishers need to implement measures that guarantee the reproducibility of computational models and analysis tools. The establishment of reproducible analyses and modelling records along with *every* publication is a means to strengthen self-correcting mechanisms in the scientific community. As an example, authors of PLOS journals are expected to make all relevant software available. Additionally, they are responsible that the software remains usable, regardless of versions or upgrades. This can be ensured using MAGPIE as a versioning system. As a second example, authors for publications in Nature journals are encouraged to deposit models in SBML repositories. However, a platform to upload and execute models in *any* programming language will establish an even more transparent review process by enabling reviewers to execute models in the browser without local installation. After publication, it can be expected that readily available models will increase visibility and citation of associated publications, and also ensure the reproducibility of research findings.

### Availability

MAGPIE itself adheres to the standards for reproducible research [4]. The source code of the latest server application can be downloaded from the GitHub repository at https://github.com/christbald/magpie. Furthermore, the source code at the time of publication can be found at https://zenodo.org/record/1044034.

The corresponding R package, providing a remote access to MAGPIE, is available at https://github.com/christbald/magpie_api_r.

A demo server can be accessed at https://magpie.imb.medizin.tu-dresden.de. Within the demo server, the computation time per job is restricted to 1 hour and the memory usage to 2 GB RAM.
