# Correction: MAGPIE: Simplifying access and execution of computational models in the life sciences

**DOI:** 10.1371/journal.pcbi.1006083

**Published:** 2018-03-26

**Authors:** The PLOS Computational Biology Staff

The acknowledgments for this article should read as follows:

“We thank Dr. Hans H. Diebner, who advised us on the concepts of Star and Griesemer, who already in the late 80s identified the problems of interdisciplinary work and developed the theoretical concept of boundary objects.”

## References

[1] Baldow C, Salentin S, Schroeder M, Roeder I, Glauche I (2017) MAGPIE: Simplifying access and execution of computational models in the life sciences. PLoS Comput Biol 13(12): e1005898 https://doi.org/10.1371/journal.pcbi.1005898 29244826 10.1371/journal.pcbi.1005898 PMC5747461

